# Patterns of Fertility Preservation and Pregnancy Outcome After Breast Cancer at a Large Comprehensive Cancer Center

**DOI:** 10.1089/jwh.2018.6986

**Published:** 2019-04-22

**Authors:** Maria Vittoria Dieci, Cristina Ghiotto, Caterina Barbieri, Gaia Griguolo, Carlo Saccardi, Michele Gangemi, Alfonso Pluchinotta, Elisabetta Di Liso, Carlo Alberto Giorgi, Tommaso Giarratano, Giulia Tasca, Grazia Vernaci, Giovanni Faggioni, Pierfranco Conte, Valentina Guarneri

**Affiliations:** ^1^ Department of Surgery, Oncology and Gastroenterology, University of Padova, Padova, Italy.; ^2^ Medical Oncology 2, Istituto Oncologico Veneto IRCCS, Padova, Italy.; ^3^ Department of Woman and Child Health, University of Padova, Padova, Italy.; ^4^ Department of Surgery-Breast Surgery, Policlinico of Abano Terme, Padova, Italy.

**Keywords:** cancer survivorship, fertility counseling, breast cancer, fertility preservation, pregnancy after breast cancer

## Abstract

***Background:*** In the last decades, long-term outcomes of breast cancer (BC) patients have improved, raising new survivorship issues, including fertility preservation and safety of pregnancy after BC. This study assesses evolution in patterns of fertility discussion/preservation over time and reports pregnancy outcomes in a cohort of young BC patients.

***Methods:*** A retrospective cohort of 590 BC patients aged ≤40 diagnosed between 2000 and 2016 at a large cancer center was identified. Fertility counseling and preservation patterns for patients receiving chemotherapy were analyzed and compared for two cohorts: 2004–2006 and 2014–2016 (total *n* = 161). Outcomes were reported for patients with documented pregnancy after BC.

***Results:*** Significantly, more patients diagnosed in 2014–2016 had evidence of discussion on fertility issues and/or application of fertility preservation techniques versus patients diagnosed in 2004–2006 (82.9% vs. 66.0%, *p* = 0.017). In particular, there was a significant difference in rate of documented fertility issues discussion (67.6% vs. 34.0%, *p* < 0.001). Age >35 and parity were associated with lower rates of fertility discussion/preservation. However, rates significantly improved over time (77.6% in 2014–2016 vs. 58.1% in 2004–2006 for patients aged >35, *p* = 0.046; 80.7% in 2014–2016 vs. 57.6% in 2004–2006 for patients with children at diagnosis, *p* = 0.018). Twenty-six patients with pregnancy after BC were identified; eight delivered at the age of >40. No complications for women or newborns were reported. Only two patients experienced BC relapse.

***Conclusions:*** In this small retrospective cohort, no safety concerns were identified for pregnancy after BC. The importance attributed by clinicians to address fertility issues has increased over time.

## Introduction

Breast cancer (BC) is the most common malignancy in women, accounting for 1.67 million new cancer cases diagnosed in 2012 worldwide,^1^ with more than 10% of new cases diagnosed in women younger than the age of 40 years (more than 190,000 new cases estimated worldwide in 2012).^2^ BC is also the most common malignancy in women in Italy, with more than 50,000 estimated new cases in 2017, and accounts for almost half (41%) of the malignancies diagnosed in women younger than the age of 50 years.^3^

In the last decades, BC mortality has consistently decreased,^1^ thanks to the extensive use of screening and advances in adjuvant systemic treatments. However, both chemotherapy and endocrine treatment may affect the reproductive function of young BC patients. As the number of BC survivors increases, the need to preserve fertility in young BC patients and their potential desire to build or complete their family beyond the diagnosis of BC is becoming a major issue.

Pregnancy after BC has been discouraged for a long time in the belief that increased estrogen levels during gestation may promote the growth of micrometastatic disease, favoring BC relapse. However, recent studies have shown that pregnancy after BC diagnosis is safe, contradicting the initial position. In the large retrospective study by Azim et al., no difference in disease-free survival was described between BC patients who became pregnant (*n* = 333) and a matched cohort of controls (*n* = 874); results were similar according to estrogen receptor status of primary BC.^4,5^

On these bases, current guidelines do not recommend abortion in case of pregnancy after BC diagnosis.^6–8^

The increased awareness of the safety of pregnancy after BC should influence clinician's sensibility toward fertility issues, calling for a careful management and comprehensive discussion with young BC patients.

This work was conducted retrospectively at a large cancer Institution (Istituto Oncologico Veneto, Padova, Italy), with two main aims:

- to assess time-dependent variations in fertility preservation for young BC patients
- to describe characteristics, BC outcome and pregnancy outcome of consecutive patients who became pregnant after BC diagnosis.

## Methods

An Institutional Review Board-approved chart review was performed on all patients diagnosed with primary nonmetastatic BC at the age of 40 years or younger who were referred at the Istituto Oncologico Veneto o Padova (Italy) between 2000 and 2016. Due to the limited possibilities of the success of fertility preservation techniques in women older than 40 years of age,^9^ we focused our attention only on women aged 40 years or younger.

To assess time-dependent changes in patterns of fertility preservation, young patients who received adjuvant or neoadjuvant chemotherapy were identified and two cohorts were considered: patients diagnosed in the years 2004–2006 and patients diagnosed in the years 2014–2016. Collected data included age at diagnosis, clinicopathological tumor features, type of systemic treatment, parity at diagnosis, documentation in medical records of fertility issues discussion, and fertility preservation method. All data were retrospectively extracted from medical records, including multidisciplinary meeting reports, and were collected in a dedicated database.

Next, we focused on the cohort of patients diagnosed with BC at the age of 40 or younger between 2000 and 2016 who had documentation in clinical records of pregnancy thereafter. For these patients, the following data were collected: age at diagnosis, tumor stage, hormone receptor (HR) and human epidermal growth factor receptor 2 (HER2) status, type of surgery, type and duration of systemic treatment, radiotherapy, fertility preservation, delivery date, age at delivery, pregnancy outcome, breastfeeding, subsequent pregnancies, abortions, and oncologic follow-up.

### Statistical analysis

Statistical analysis was performed with SPSS Version 24.

All the analyses, unless otherwise specified, were descriptive in nature. Descriptive statistics included, as appropriate, frequency counts and percentages in contingency tables, medians and ranges, concordance percentages, and graphical displays such as bar charts. The chi-square test was used to evaluate the associations between variables.

Disease-free survival from BC diagnosis was defined as the time interval from BC diagnosis to locoregional/distant relapse, second primary invasive BC, and death from any cause (whichever occurred first).

Disease-free survival from delivery was calculated as the time interval from the date of delivery to locoregional/distant relapse, second primary invasive BC, and death from any cause (whichever occurred first).

Survival curves were estimated according to the Kaplan–Maier method. All hypothesis tests were conducted at a two-sided alpha level of 0.05.

## Results

Overall, medical records of more than 9,000 patients with a diagnosis of primary BC between 2000 and 2016 were reviewed, and *n* = 590 patients (around 6% of the total) aged 40 years or younger at diagnosis were identified (Supplementary Fig. S1).

### Patterns of fertility issues discussion and fertility preservation among young BC patients

To assess variations in fertility preservation patterns over time, we considered young patients who received adjuvant or neoadjuvant chemotherapy and who were diagnosed in years 2004–2006 (*n* = 50, 6% of total patients diagnosed in that period) or in years 2014–2016 (*n* = 111, 6% of total patients diagnosed in that period) (Supplementary Fig. S1). Patient characteristics are reported in Table 1.

**Table 1.** Summary of Patient Demographics and Tumor Characteristics

	*Cohort 2004–2006*		*Cohort 2014–2016*	
	N *(total = 50)*	*%*	N *(total = 111)*	*%*
Median age at diagnosis (range)	36 (26–40)		36 (23–40)	
Age at diagnosis, years				
≤35	19	38.0	44	39.6
>35	31	62.0	67	60.4
Parity				
No	13	27.0	44	42.7
Yes	33	68.8	57	55.3
BC diagnosis during pregnancy	2	4.2	2	2
Histotype				
Ductal	40	80.0	104	93.7
Lobular	5	10.0	3	2.7
Other	5	10.0	4	3.6
Stage at diagnosis				
I	18	36.0	22	19.8
II	16	32.0	62	55.9
III	16	32.0	27	24.3
ER status				
Positive (≥10%)	36	72.0	70	63.1
Negative	14	28.0	41	36.9
PgR status				
Positive (≥10%)	31	62.0	51	45.9
Negative	19	38.0	60	54.1
HR status				
Positive (ER and/or PgR >10%)	38	76.0	71	64.0
Negative (ER and PgR <10%)	12	24.0	40	36.0
HER2				
Positive	15	30.0	29	26.1
Negative	35	70.0	82	73.9
Adjuvant HT				
Yes	38	76.0	69	62.7
No	12	24.0	41	37.3
Documentation of fertility counseling				
Yes	17	34.0	75	67.6
No	33	66.0	36	32.4
Fertility preservation technique				
GnRH analog	31	62.0	72	64.9
GnRH analog+oocyte cryopreservation	0	0.0	10	9.0
None	19	38.0	29	26.1
Documentation in medical records of fertility counseling and/or technique				
Yes	33	66.0	92	82.9
No	17	34.0	19	17.1

BC, breast cancer; ER, estrogen receptor; GnRH, gonadotropin-releasing hormone; HER2, human epidermal growth factor receptor 2; HR, hormone receptor; HT, hormonal therapy; PgR, progesterone receptor.

**FIG. 1.**
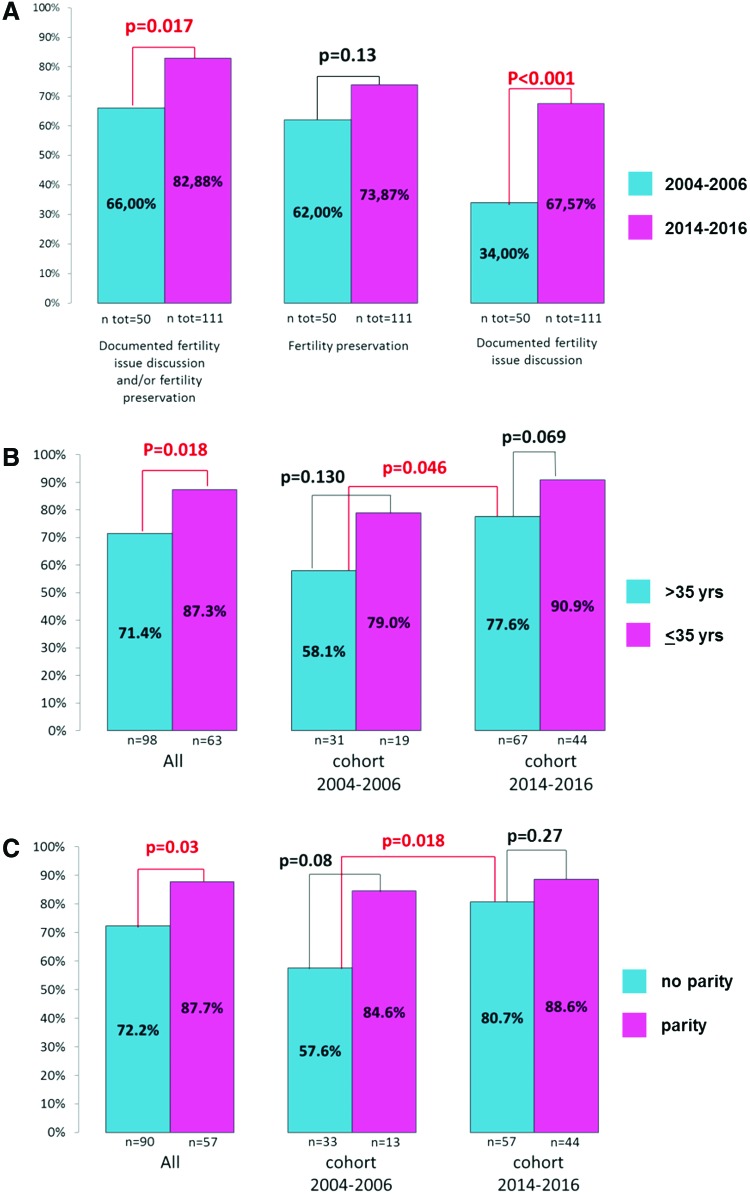
Proportion of patients with documented fertility issue discussion and/or fertility method application according to clinical characteristics and time period. Proportion of patients with documented fertility issue discussion and/or fertility method application in relationship to different time cohorts **(A)**; proportion of patients with documented fertility issue discussion and/or fertility method application in relationship to different time cohorts by age **(B)**; proportion of patients with documented fertility issue discussion and/or fertility method application in relationship to different time cohorts by parity **(C)**. Color images are available online.

Overall, for 77.6% out of 161 total patients, there was evidence in medical records of discussion on fertility issues related to chemotherapy and/or application of fertility preservation methods, with significant difference when comparing the two time cohorts (82.9% of patients diagnosed in years 2014–2016 vs. 66.0% of patients diagnosed in 2004–2006, *p* = 0.017), as shown in Figure 1A. When considering fertility preservation rate alone, no significant difference between the two time cohorts was observed, although it was numerically higher in the more recent cohort (73.9% vs. 62.0%, *p* = 0.13, Fig. 1A). However, there was a significant difference in the frequency of documentation of fertility issues discussion according to time cohort: 75 out of 111 (67.6%) patients diagnosed in years 2014–2016 had documentation in medical records of discussion of fertility issues versus 17 (34.0%) of the 50 patients diagnosed in years 2004–2006 (*p* < 0.001). Overall, the most frequent fertility method was gonadotropin-releasing hormone (GnRH) analog administration concomitant to chemotherapy (*n* = 103 of 113 total patients), while oocyte cryopreservation (followed by GnRH analog administration) was used in 10 patients (all of which in the 2014–2016 cohort).

We then investigated whether factors such as age at diagnosis and parity may have affected fertility discussion/preservation patterns. Patients aged 35 years or younger were more likely to have fertility discussion or to undergo fertility preservation compared with older patients, overall and in the two time cohorts separately, as shown in Figure 1B. The proportion of patients aged >35 years with documentation of fertility discussion/preservation was significantly higher in the more recent cohort versus the less recent cohort (77.6% vs. 58.1%, *p* = 0.046). As shown in Figure 1C, parity before BC diagnosis was also associated with a lower rate of fertility discussion documentation/preservation, overall and in the two time cohorts separately; however, in the more recent cohort the proportion of patients with documented fertility discussion/preservation was significantly higher compared to the less recent cohort (80.7% vs. 57.6%, *p* = 0.018).

### Outcome of patients with pregnancy after BC diagnosis

Among 590 patients diagnosed with BC at the age of 40 years or younger between 2000 and 2016, 26 cases presented documentation of pregnancy after BC diagnosis (4.4%, Supplementary Fig. S1). Table 2 summarizes demographics, tumor characteristics, and treatments of these 26 patients. Median age at diagnosis was 32 (range 27–40). The majority of the patients had tumors of ductal histology with positive HR status and moderate/high tumor grade. Eighteen patients received adjuvant and/or neoadjuvant chemotherapy, with concomitant GnRH agonist in 61% (*n* = 11) of these cases. A majority of patients (*n* = 15) received endocrine adjuvant treatment; among them, five did not complete the planned 5 years of treatment due to: voluntary treatment interruption or adverse events (*n* = 3), depression related to the fear of never being able to become pregnant (*n* = 1), and pregnancy during treatment (*n* = 1).

**Table 2.** Patients with Pregnancy After Breast Cancer Diagnosis: Summary of Patient Demographics, Tumor Characteristics, and Treatment Received

	N *(total = 26)*	*%*
Median age at diagnosis (range)	26	32 (27–40)
Parity at BC diagnosis		
0	14	63.6
1	5	22.7
2	2	9.1
BC diagnosis during pregnancy	1	4.5
Missing	4	
Histotype		
Ductal	17	77.3
Lobular	1	4.5
Other	4	18.2
Missing	4	
Stage at diagnosis		
I	10	40
II–III	15	60
Missing	1	
Grade		
I	3	15
II	9	45
III	8	40
Missing	6	
HR		
Positive (ER and/or PgR ≥10%)	18	78.3
Negative (ER and PgR <10%)	5	21.7
Missing	3	
Ki67%, median (range)	21	20 (2–85)
Adjuvant/neoadjuvant CT		
Yes	18	72
No	7	28
Missing	1	
If CT, GnRH analog for fertility preservation, *n* (total = 18)	11	61.1
Adjuvant HT		
Yes	15	57.7
No	11	42.3

CT, chemotherapy.

Table 3 reports data on pregnancy and delivery outcome. Overall, 38 pregnancies after BC diagnosis were reported: 30 deliveries (4 patients had 2 subsequent successful pregnancies after BC diagnosis) and 8 spontaneous abortions. Four patients had one spontaneous abortion each before the first successful pregnancy after BC diagnosis. The other four abortions occurred after the first successful pregnancy post-BC in two patients (one patient with three abortions). At least one successful pregnancy was reported for each patient included in this cohort. All pregnancies were achieved without the use of medically assisted procreation techniques. Median age at first delivery was 38 years, ranging from 32 to 44 years, with eight patients who delivered at the age of 40 or older.

**Table 3.** Patients with Pregnancy After Breast Cancer: Summary of Data Regarding Pregnancy and Delivery Outcome

*First delivery after diagnosis*	n	*%*
Patients, *N* total	26	100
Cesarean delivery	6	30.0
Age at first delivery, years: median (range)	38 (32–44)	
Newborns, *N*	27	100
Female	15	51.6
Male	8	29.0
Missing	4	19.4
Gestational week, median (range)	40 (38–41)	

*Second delivery after diagnosis*	n
Patients	4
Newborns	4

*Spontaneous abortions after diagnosis*	n
Patients	6
Abortions	8

*Abortions between diagnosis and first delivery*	N
Patients	4
Abortions	4

The median gestational week at delivery and weight of the newborns were within physiologic ranges and all newborns were healthy and did not present any complication. Breastfeeding was documented for five patients.

Median time from BC diagnosis to delivery was 64 months (range 19–108). Median time from last chemotherapy dose and last endocrine treatment dose to delivery was 72 (16–96) and 22 (6–50) months, respectively.

Median follow-up from diagnosis was 9.4 years (95% CI 7.9–10.9 years) and median follow-up from delivery was 3.8 years (95% CI 1.5–6.1 years). Two disease-free survival (DFS) events were observed (BC relapse). DFS rates at 10 years after diagnosis and at 5 years after delivery were 93% and 92%, respectively.

## Discussion

Over the past decade, there has been an increasing trend of women delaying childbearing,^10^ which, in addition to a continuous decline in recurrence rates and risk of death secondary to BC,^1^ has led physicians to give increasingly more attention to fertility issues in BC patients.

In this retrospective analysis, we identified a cohort of 590 young patients diagnosed with invasive BC between 2000 and 2016. In 26 cases (4.4%) medical records reported successful pregnancy after diagnosis, which is consistent with literature data.^11^ No case of preterm delivery or fetal malformation was reported. This data confirms that young BC survivors may manage to become pregnant, emphasizing the need to improve patient counseling on this subject.^11,12^ This need is additionally stressed by the observation that two patient in this cohort stopped endocrine therapy prematurely due to pregnancy related issues (one for depression related to the fear of never being able to become pregnant and one due to pregnancy during treatment). At this time, there is insufficient data to draw formal recommendations on correct timing of pregnancy after BC and consequent management of adjuvant treatments interruptions/resumptions. An ongoing trial (NCT02308085), which is investigating the safety of temporarily interrupting endocrine therapy, with the goal to permitting pregnancy, will help physicians collect evidence to adequately respond to patients on these issues. Due to the limited sample size of our cohort and to the absence of a control group, data on pregnancy after BC are exploratory and descriptive in nature. Indeed, the risk of selection bias called “health mother effect” could not be controlled in our study. Even so, it is reassuring that our experience appears somehow consistent with more recent evidence from large cohorts reporting that pregnancy does not increase risk of BC relapse, even in HR+ BC patients,^4,5^ and that newborns are not exposed to higher risk of prenatal/perinatal complications.^13^

Interestingly, observing the characteristics of patients who successfully became pregnant after BC may also help dispel some presumptions regarding which patients should undergo fertility preservation and fertility preservation counseling. In fact, both age and parity are well-known barriers to fertility preservation counseling as clinicians may be induced to assume that patients have completed child-bearing in the first case, or have fully satisfied their desire for family in the second case.^14–16^ In our cohort of patients who successfully mothered children after BC, we observed 7 patients who already had children at time of diagnosis, and patients as old as 40 years at diagnosis, thus pointing out that motherhood after cancer might not be a prerogative of extremely young patients nor a desire reserved to childless women.

This leads to the other aim of the study, which was to evaluate physician awareness regarding fertility issues by assessing time-dependent changes in a 10-year period in fertility preservation counseling and techniques in young BC patients undergoing neo/adjuvant chemotherapy.

In both time cohorts (2004–2006 and 2014–2016), the majority of BC patients aged ≤40 years had evidence in medical records of discussion on fertility issues and/or application of fertility preservation methods. This compares favorably with published data reporting a counseling rate of around 26% in BC patients.^14,16^ Nevertheless, the most recent cohort showed a significantly higher rate (82.9% vs. 66.0%, *p* = 0.017) of fertility counseling documentation and/or application of fertility preservation methods. This difference is mostly due to an increase in the documentation of fertility counseling (67.6% vs. 34.0%, *p* = 0.06).

Even if counseling does not finally translate in the application of fertility preservation techniques, its documentation in medical records is not irrelevant. Actually, it reflects physicians' awareness and the importance attributed to addressing fertility issues before initiation of treatment, independently from the decision taken by the patient. Clinical data show that female survivors who previously received fertility counseling have less regret and improved quality of life, independently from the final decision on pursuing or not the fertility preservation.^17^

In our study, the proportion of patients aged >35 years and/or who already had children receiving counseling was significantly higher in the more recent cohort versus the older cohort, highlighting progressive increase in clinician's perceived importance of extending counseling to all young BC patients. These modifications in clinical practice adequately mirror the evolution of guidelines, as depicted by the 2013 ASCO recommendation that no patient should be excluded from fertility preservation discussion for any reason, including age, prognosis, socioeconomic status, or parity.^18,19^

In the most recent cohort (2014–2016), 9.0% of patients finally underwent oocyte cryopreservation procedures, an acceptance rate similar to the one reported by Ruddy et al. in 2014.^20^ However, in both the 2004–2006 cohort and the 2014–2016 cohort, more than 60% of patients received an GnRH analog during chemotherapy. This is in keeping with the general attitude of Italian oncologists: in a recent survey, aimed to investigate the approach of Italian oncologists and breast surgeons dealing with BC to fertility issues, 65% of panelists declared to use concomitant administration of GnRH analogs and chemotherapy regularly in their common clinical practice.^21^ This attitude might be influenced by the fact that the PROMISE trial, a large randomized trial concerning the use of GnRH analogs in this setting, was conducted in Italy.^22^ Moreover, this option is also recommended by national oncological guidelines.^23^

## Conclusions

Physicians' attention to fertility issues and counseling has increased over time, also regarding women who already have children and older patients.

In this small retrospective cohort, no safety concerns were identified for pregnancy after BC. However, due to the limited sample size of our cohort and to the absence of a control group, data on pregnancy after BC are only descriptive, as risk of selection bias could not be controlled. Even so, this evidence appears consistent with data from large cohorts reporting that pregnancy does not increase risk of BC relapse, even in HR+ BC patients.^4,5^ Nevertheless, there are insufficient data to draw formal recommendations on correct timing of pregnancy after BC, and ongoing trials are investigating the best management of adjuvant treatments' interruptions/resumptions.

This analysis contributes in stressing the importance of adequate fertility issue discussion and counseling for young BC patients and the need to keep reducing barriers in the field.

## Ethical Approval

All procedures performed in studies involving human participants were in accordance with the ethical standards of the institutional and/or national research committee and with the Declaration of Helsinki 1964 and its later amendments or comparable ethical standards. The study was approved by the local Ethics Committee. For this type of study, formal consent is not required.

## Supplementary Material

Supplemental data

## References

[1] Torre LA, Bray F, Siegel RL, Ferlay J, Lortet-Tieulent J, Jemal A Global cancer statistics, 2012. CA Cancer J Clin 2015;65:87–108 25651787 10.3322/caac.21262

[2] Fidler MM, Gupta S, Soerjomataram I, Ferlay J, Steliarova-Foucher E, Bray F Cancer incidence and mortality among young adults aged 20–39 years worldwide in 2012: A population-based study. Lancet Oncol 2017;18:1579–1589 29111259 10.1016/S1470-2045(17)30677-0

[3] AIRTUM-AIOM. The numbers of cancer in Italy 2017. Il Pensiero Scientifico Editore, 2017

[4] Azim HA Jr., Kroman N, Paesmans M, et al. Prognostic impact of pregnancy after breast cancer according to estrogen receptor status: A multicenter retrospective study. J Clin Oncol 2013;31:73–79 23169515 10.1200/JCO.2012.44.2285 PMC3530692

[5] Lambertini M, Kroman N, Ameye L, et al. Long-term safety of pregnancy following breast cancer according to estrogen receptor status. J Natl Cancer Inst 2018;110:426–429 29087485 10.1093/jnci/djx206 PMC6658852

[6] Cardoso F, Loibl S, Pagani O, et al. The European Society of Breast Cancer Specialists recommendations for the management of young women with breast cancer. Eur J Cancer 2012;48:3355–3377 23116682 10.1016/j.ejca.2012.10.004

[7] Peccatori FA, Azim HA Jr, Orecchia R, et al. Cancer, pregnancy and fertility: ESMO Clinical Practice Guidelines for diagnosis, treatment and follow-up. Ann Oncol 2013;24(Suppl 6):vi160–vi170 23813932 10.1093/annonc/mdt199

[8] Paluch-Shimon S, Pagani O, Partridge AH, et al. ESO-ESMO 3rd international consensus guidelines for breast cancer in young women (BCY3). Breast 2017;35:203–217 28822332 10.1016/j.breast.2017.07.017

[9] Lambertini M, Anserini P, Levaggi A, Poggio F, Del Mastro L Fertility counseling of young breast cancer patients. J Thorac Dis 2013;5(Suppl 1):S68–S80 23819030 10.3978/j.issn.2072-1439.2013.05.22 PMC3695540

[10] Matthews TJ, Hamilton BE Delayed childbearing: More women are having their first child later in life. NCHS Data Brief 2009;21:1–8 19674536

[11] Litton JK Breast cancer and fertility. Curr Treat Options Oncol 2012;13:137–145 22396154 10.1007/s11864-012-0185-5

[12] Letourneau JM, Smith JF, Ebbel EE, et al. Racial, socioeconomic, and demographic disparities in access to fertility preservation in young women diagnosed with cancer. Cancer 2012;118:4579–4588 22451228 10.1002/cncr.26649 PMC3387319

[13] Jacob L, Kalder M, Arabin B, Kostev K Impact of prior breast cancer on mode of delivery and pregnancy-associated disorders: A retrospective analysis of subsequent pregnancy outcomes. J Cancer Res Clin Oncol 2017;143:1069–1074 28220257 10.1007/s00432-017-2352-3 PMC11818950

[14] Quinn GP, Block RG, Clayman ML, et al. If you did not document it, it did not happen: Rates of documentation of discussion of infertility risk in adolescent and young adult oncology patients' medical records. J Oncol Pract 2015;11:137–144 25549654 10.1200/JOP.2014.000786 PMC4799849

[15] Penrose R, Beatty L, Mattiske J, Koczwara B Fertility and cancer—A qualitative study of Australian cancer survivors. Support Care Cancer 2012;20:1259–1265 21660668 10.1007/s00520-011-1212-y

[16] McCray DK, Simpson AB, Flyckt R, et al. Fertility in women of reproductive age after breast cancer treatment: Practice patterns and outcomes. Ann Surg Oncol 2016;23:3175–3181 27334218 10.1245/s10434-016-5308-y

[17] Letourneau JM, Ebbel EE, Katz PP, et al. Pretreatment fertility counseling and fertility preservation improve quality of life in reproductive age women with cancer. Cancer 2012;118:1710–1717 21887678 10.1002/cncr.26459 PMC3235264

[18] Lee SJ, Schover LR, Partridge AH, et al. American Society of Clinical Oncology recommendations on fertility preservation in cancer patients. J Clin Oncol 2006;24:2917–2931 16651642 10.1200/JCO.2006.06.5888

[19] Loren AW, Mangu PB, Beck LN, et al. Fertility preservation for patients with cancer: American Society of Clinical Oncology clinical practice guideline update. J Clin Oncol 2013;31:2500–2510 23715580 10.1200/JCO.2013.49.2678 PMC5321083

[20] Ruddy KJ, Gelber SI, Tamimi RM, et al. Prospective study of fertility concerns and preservation strategies in young women with breast cancer. J Clin Oncol 2014;11:1151–1156 10.1200/JCO.2013.52.8877 PMC4164759 24567428

[21] Biglia N, Torrisi R, Codacci Pisanelli G, Rota S, Peccatori FA Gynecological endocrinology attitudes on fertility issues in breast cancer patients: An Italian survey attitudes on fertility issues in breast cancer patients: An Italian survey. Gynecol Endocrinol 2015;31:458–464 25982361 10.3109/09513590.2014.1003293

[22] Del Mastro L, Boni L, Michelotti A, et al. Effect of the gonadotropin-releasing hormone analogue triptorelin on the occurrence of chemotherapy-induced early menopause in premenopausal women with breast cancer. JAMA 2011;306:269–276 21771987 10.1001/jama.2011.991

[23] Lambertini M, Cinquini M, Moschetti I, et al. Temporary ovarian suppression during chemotherapy to preserve ovarian function and fertility in breast cancer patients: A GRADE approach for evidence evaluation and recommendations by the Italian Association of Medical Oncology. Eur J Cancer 2017;71:25–33 27940355 10.1016/j.ejca.2016.10.034

