# Supplementary figures and images for: Patterns of Fertility Preservation and Pregnancy Outcome After Breast Cancer at a Large Comprehensive Cancer Center
Source: J Womens Health (Larchmt). 2019 Apr 22;28(4):544–50. doi: 10.1089/jwh.2018.6986 (PMC6482901; doi:10.1089/jwh.2018.6986)

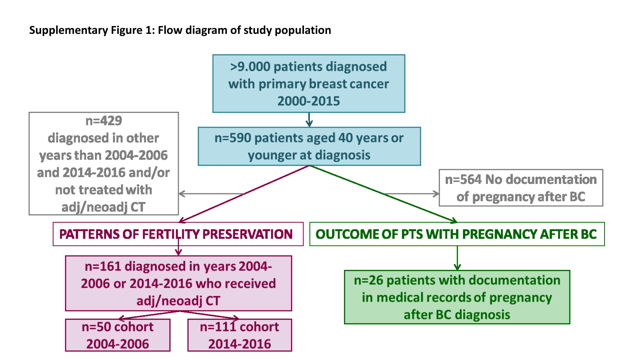

Supplement: Supplemental data [file Supp_Fig1.tif]
